# Dataset on enhanced magnetic refrigeration capacity in Ni–Mn–Ga micro-particles

**DOI:** 10.1016/j.dib.2018.05.053

**Published:** 2018-05-17

**Authors:** Mingfang Qian, Xuexi Zhang, Zhenggang Jia, Xinhao Wan, Lin Geng

**Affiliations:** School of Materials Science and Engineering, Harbin Institute of Technology, Harbin 150001, China

## Abstract

The dataset presented in this paper is supporting the research article “Enhanced magnetic refrigeration capacity in Ni-Mn-Ga micro-particles” (Qian, et al., 2018) [1]. The martensite transformation temperature (*M*_*s*_) and the Curie point (*T*_*c*_) of the annealed alloys with nominal composition Ni_55-x_Mn_20+x_Ga_25_ (*x* = 0, 0.25, 0.5, 1, atomic percent, labeled as A1, A2, A3 and A4, respectively) varied with x, yielding a temperature difference *T*_*c*_-*M*_*c*_ of 7.2 K at *x* = 0.5 (A3). The magnetization difference (*ΔM*) between the austenite and martensite, the field dependence of transformation temperature (*ΔT*/*ΔH*) and the thermal hysteresis loss of A3 and the according stress relief annealing (SRA) particles were demonstrated. The isothermal magnetization curves of A3 and the SRA particles were measured in order to determine the magnetocaloric effect.

**Specifications Table**

**Table:** 

Subject area	*Physics*
More specific subject area	Materials Science
Type of data	*Table, image*
How data was acquired	*TA Q2000 differential scanning calorimeter (DSC); Vibrating sample magnetometer (VSM) in a Quantum Design Physical Property Measurement System (PPMS), Database analysis*
Data format	*Raw, analyzed*
Experimental factors	*Ni-Mn-Ga alloy ingots with nominal composition of Ni*_*55-x*_*Mn*_*20+x*_*Ga*_*25*_*(x = 0, 0.25, 0.5, 1, atomic percent) were prepared by induction melting and were re-melted four times. The ingots were annealed at 1173 K for 24 h, then furnace cooled to room temperature. The annealed x = 0.5 ingot was crashed into fragments. Particles were prepared by manually grinding the fragments and subsequently screened by sieves. The particles were annealed at 773 K for 20 h, and then furnace cooled to room temperature.*
Experimental features	*The martensite transformation temperature of the bulk alloys were examined by DSC. The isofield magnetization curves (M-T) were tested in the VSM and the according ΔM, ΔT*/*ΔH, thermal hysteresis losses and the T*_*c*_ of A2 *were assessed based on the M-T curves. The Isothermal magnetization curves (M-H) curves were recorded by the PPMS.*
Data source location	*Harbin, China*
Data accessibility	*data is with this article*

**Value of the data**

• This data fulfills the background of the research article in choosing the nominal composition of Ni_54.5_Mn_20.5_Ga_25.0_.
• This data will be useful in understanding the magnetocaloric properties of both bulk alloy and single crystalline micron-sized particles.
• This data provides raw data for magnetic entropy change calculation for both bulk alloy and SRA particles.

## 1 Data

**Fig. 1:**
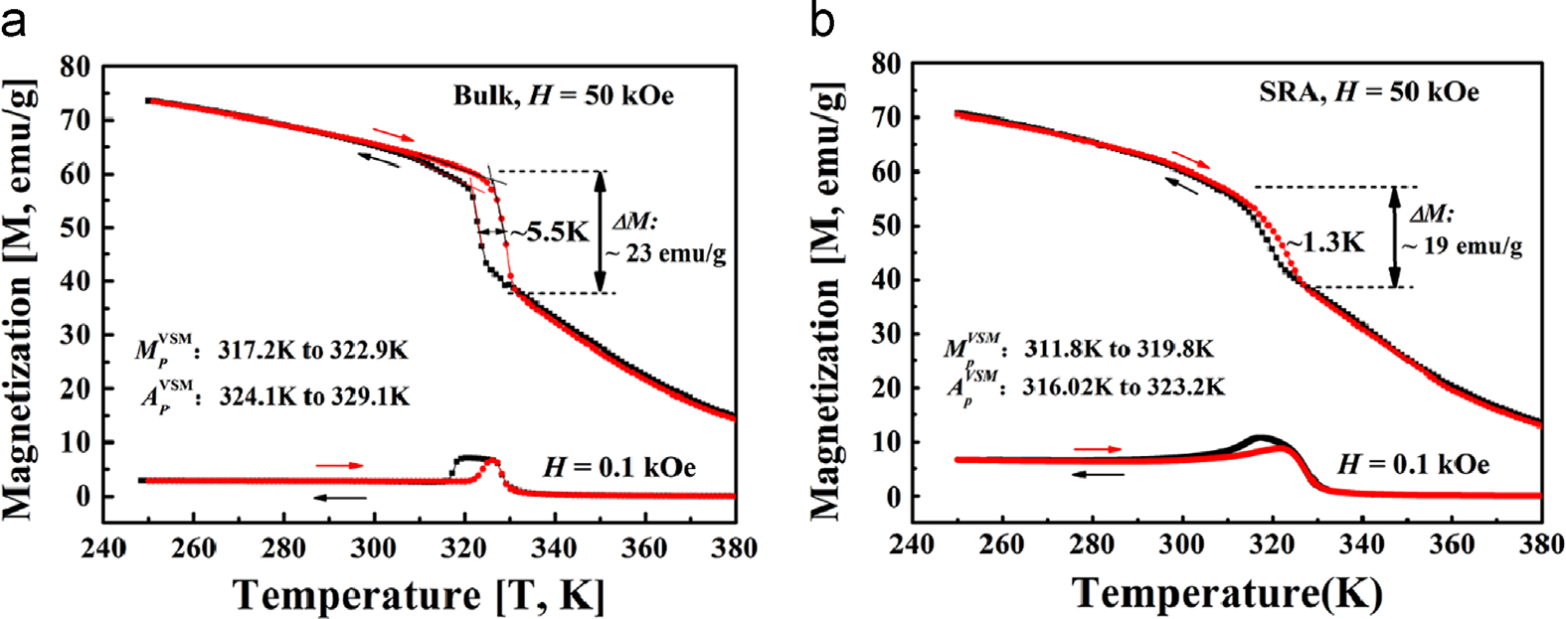
Isofield magnetization curves (*M-T*) curves recorded at magnetic fields *H* = 0.1 and 50 kOe upon cooling and heating in (a) Ni-Mn-Ga bulk alloy A3 and (b) stress relief annealing (SRA) particles.

**Fig. 2:**
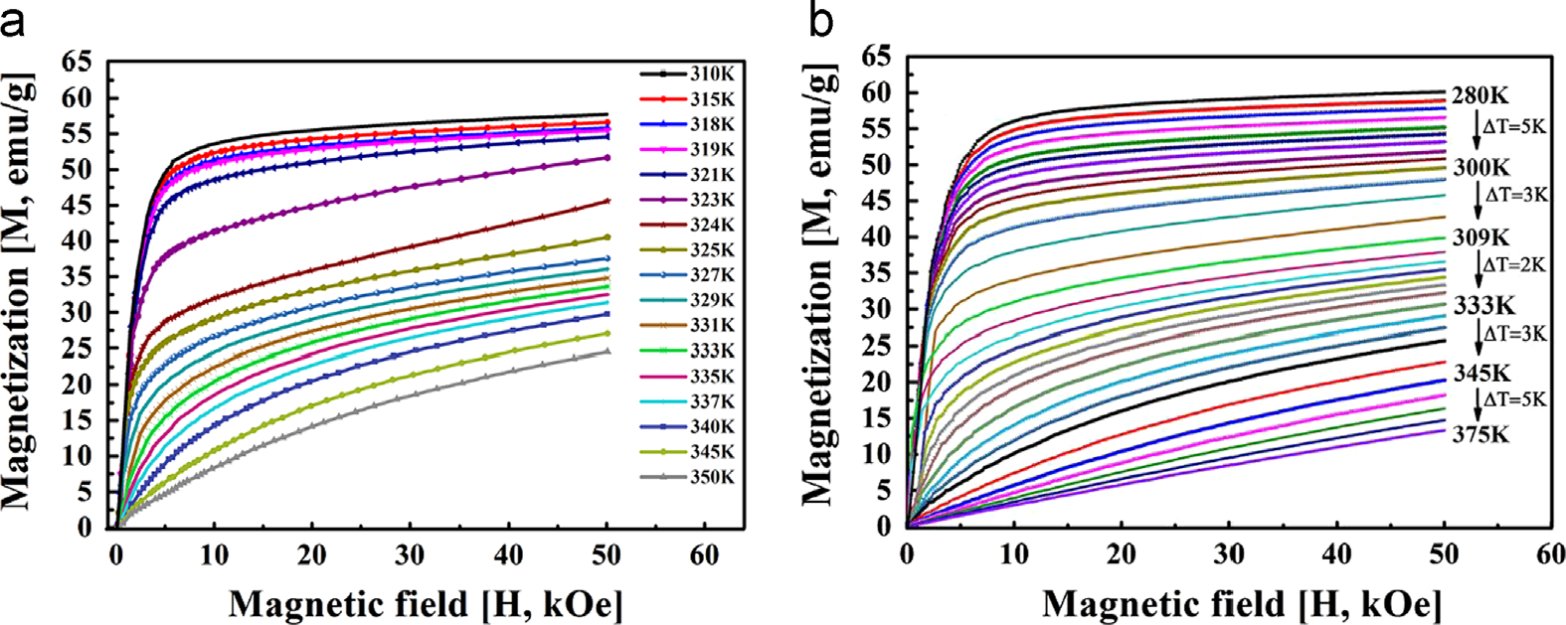
Isothermal magnetization curves (*M-H*) of (a) bulk alloy A3 in the temperature range from 310 to 350 K and (b) stress relief annealing (SRA) particles in the temperature range from 280 to 375 K.

This dataset summarizes the martensite transformation (MT) temperatures, *M*_*s*_, *M*_*f*_, *A*_*s*_, *A*_*f*_, the Cuire point *T*_*c*_ and the absolute latent heat, |*ΔL*|, of the bulk Ni_55-x_Mn_20+x_Ga_25_ (x = 0, 0.25, 0.5, 1) alloys based on DSC curves, as shown in Table 1. The dataset also provides the martensite transformation, thermal hysteresis losses, magnetic properties of the bulk alloy (A3) and the according stress relief annealing (SRA) particles. Fig. 1 displays the low field (0.1 kOe) and high field (50 kOe) isofield magnetization curves (*M-T*) of the A3 and SRA particles, respectively. Table 2 summarizes *A*_*p*_^*VSM*^ and *M*_*p*_^*VSM*^ (maximum of the 1st derivative of the *M-T* curves during heating and cooling, respectively) shifting upon the field variation of 50 kOe based on Fig. 1. Fig. 2 shows the isothermal magnetization curves (*M-H*) of the bulk alloy A3 and the SRA particles, respectively.

**Table 1:** Martensite transformation temperature and latent heat of bulk Ni_55-x_Mn_20+x_Ga_25_ (x = 0, 0.25, 0.5, 1) alloys based on DSC and VSM measurements.

Alloys	*M*_*s*_	*M*_*f*_	*A*_*s*_	*A*_*f*_	*T*_*c*_^*cooling*^	|*T*_*c*_ - *M*_*s|*_|	|*ΔL*|_heating_	|*ΔL*|_cooling_
	K						J/g	
A1, *x* = 0	267.5	263.4	268.4	272.6	316.8	49.3	4.9	4.9
A2, *x* = 0.25	348.9	340.8	350.9	358.0	347.4(VSM)	1.5	8.4	8.1
A3, *x* = 0.5	317.5	312.2	320.0	327.6	324.7	7.2	7.3	8.4
A4, *x* = 1	303.4	298.8	302.7	308.6	335.6	32.2	4.8	5.7

**Table 2:** Transformation temperatures shift of Ni-Mn-Ga bulk alloy A3 and stress relief annealing (SRA) particles (determined by VSM).

Materials	*A*_*p*_^*VSM*^ (0.1 kOe)	*A*_*p*_^*VSM*^ (50 kOe)	*M*_*p*_^*VSM*^ (0.1 kOe)	*M*_*p*_^*VSM*^ (50 kOe)	*ΔT*_*0h*_ K	*ΔT*_*0c*_ K
Bulk alloy	324.1	329.1	317.2	322.9	5.0	5.7
SRA particle	316.0	323.2	311.8	319.8	7.2	8.0

## 2 Experimental design, materials and methods

### 2.1 Preparation of the bulk ingots

Ni-Mn-Ga alloy ingots with nominal composition of Ni_55-x_Mn_20+x_Ga_25_ (*x* = 0, 0.25, 0.5, 1, atomic percent) were prepared by induction melting pure Ni (99.99%), Mn (99.98%) and Ga (99.99%) under argon atmosphere and vacuum casting into a copper mold with a diameter of 9 mm. In order to offset the Mn evaporation during induction melting and subsequent heat treatment, 2 wt. % extra Mn was added. For homogenization, the ingots were re-melted four times and subsequently sealed in a quartz tube accompanied with Ti foils (act as oxygen getter), evacuated, annealed at 1173 K for 24 h, then furnace cooled to room temperature. The annealed alloy with nominal composition Ni_55-x_Mn_20+x_Ga_25_ (*x* = 0, 0.25, 0.5, 1, atomic percent) were labeled as A1, A2, A3 and A4, respectively.

### 2.2 Fabrication and annealing of the particles

The bulk alloy A3 was cut into small pieces with size Ф9 × 20 mm, crashed into fragments. Particles with size ~38.5–45 μm were prepared by manually grinding the fragments and subsequently screened by sieves. The particles were then sealed in a quartz tube accompanied with Ti foils, evacuated and finally annealed. A stress relief annealing (SRA) process was applied at 773 K for 20 h, and then furnace cooled to room temperature.

### 2.3 Martensite transformation of the bulk alloys

As shown in Table 1, the MT temperatures drastically increases from 267.5 K at *x* = 0 to 348.9 K at *x* = 0.25 and then decreases to 303.4 K with increasing x from 0.25 to 1, while the *T*_*c*_ values varied with *x*. A magneto-structural coupled state was attained at *x* = 0.25 where the *M*_*s*_ value 348.9 K and the *T*_*c*_ value 347.4 K (not shown on DSC curves due to the overlap with MT, obtained from *M-T* curves) are almost equal to each other, implying the overlap of the martensite transformation and the magnetic transition. With the decreasing of both MT temperatures and *T*_*c*_ at x = 0.5, a temperature difference |*T*_*c*_-*M*_*s*_| of 7.2 K was attained, which was beneficial for creating a magneto-structural partly-coupled state when a widened MT temperature range was achieved. The absolute latent heat, |*ΔL*|, shown in Table 1 was obtained by the integration of the DSC peaks. It can be found that the |*ΔL*| value of the alloys increases with decreasing |*T*_*c*_-*M*_*s*_| and varied during heating and cooling. The discrepancy during heating and cooling processes is thought to be related to the acoustic emission during MT [2].

## 3 Magnetic properties and thermal hysteresis of the stress relief annealing (SRA) particles

According to Fig. 1, it is found that the application of a magnetic field of 50 kOe leads to *A*_*p*_^*VSM*^ and *M*_*p*_^*VSM*^ temperature shift, as summarized in Table 2. The existence of the temperature shift indicated that the paramagnetic austenite can be transformed to ferromagnetic martensite under a magnetic field, i.e. the metamagnetic transformation. The temperature shift for A3 (~5 K during heating and ~5.7 K during cooling) are lower than those for the SRA particles (~7.2 K during heating and ~8.0 K during cooling). As a result, the field dependence of transformation temperature (*ΔT*/*ΔH*) was calculated to be ~0.10 K/kOe for A3 and ~0.14 K/kOe for the SRA particles during heating, which are superior to that of Ni_52_Mn_26_Ga_22_ ribbons (0.06 K/kOe) [3] and is comparable to that of Ni_55_Mn_20_Ga_25_ single crystals (0.1 K/kOe) [4].

The thermal hysteresis loss during MT transformation was also obtained from *M-T* curves under 50 kOe (demonstrated as the maximum width between the heating and cooling curves at the transition area), as shown in Fig. 1. The thermal hysteresis loss for SRA particles ~1.3 K is lower than that of the bulk alloys ~5.5 K, which is consistent with the results from the DSC curves (Fig. 3 in Ref [1]). Furthermore, as shown in Fig. 1, the near-to-saturation magnetization difference (*ΔM*) between martensite and austenite of A3 ~23 emu/g and the SRA particles ~19 emu/g were measured at 50 kOe upon heating, respectively.

M-H curves demonstrated in Fig. 2 were utilized to characterize the magnetocaloric effect. The plots were recorded under magnetic field up to 50 kOe at different temperature intervals covering both MT and magnetic transition ranges. The magnetic entropy change *ΔS*_*m*_ can be calculated from the *M-H* curves using the Maxwell relation.
